# Urban Rural Comparisons of Polycystic Ovary Syndrome Burden among Adolescent Girls in a Hospital Setting in India

**DOI:** 10.1155/2015/158951

**Published:** 2015-01-05

**Authors:** Swetha Balaji, Chioma Amadi, Satish Prasad, Jyoti Bala Kasav, Vandana Upadhyay, Awnish K. Singh, Krishna Mohan Surapaneni, Ashish Joshi

**Affiliations:** ^1^Saveetha Young Medical Researchers Group (SYMRG), Saveetha Medical College & Hospital, Faculty of Medicine, Saveetha University, Saveetha Nagar, Chennai 602105, Tamil Nadu, India; ^2^Foundation of Healthcare Technologies Society, New Delhi 110088, India; ^3^Department of Biochemistry, Saveetha Medical College & Hospital, Faculty of Medicine, Saveetha University, Saveetha Nagar, Thandalam, Chennai, Tamil Nadu 602105, India; ^4^CUNY School of Public Health, New York, NY 10035, USA

## Abstract

*Background*. Polycystic ovarian syndrome (PCOS) is a multifaceted disorder characterized by varying clinical presentations. *Objective*. The aim of this study was to determine urban and rural differences in the burden of polycystic ovarian syndrome among Indian adolescent females aged 12 to 19 years.* Methods*. A pilot cross-sectional study was conducted for a period of one month (August-September 2013) at Balaji Hospital, Vellore, Tamil Nadu, India. The final sample included 126 study participants located in various urban (50%, *n* = 63) and rural (50%, *n* = 63) settings. Information was gathered on sociodemographic and anthropometric characteristics, clinical history, occurrence of acne and hirsutism, serum testosterone levels, obstetric history, family history of chronic diseases, menstrual history, physical activity, and dietary intake. *Results*. Eighteen percent of the participants were confirmed of having PCOS by recent guidelines of Rotterdam Consensus for adolescent diagnosis of PCOS (presence of all three elements). Majority of the individuals with PCOS had an average age of 16 (SD = 2) (*P* = .02) years with an average age of menarche 12 years (SD = 1). *Conclusion*. The proportion of participants diagnosed with PCOS was higher among urban participants in comparison to rural participants.

## 1. Introduction

Polycystic ovarian syndrome (PCOS) has been defined by the National Institute of Health and Rotterdam criteria as a hormonal disorder characterized by the presence of at least one polycystic ovary (presence of multiple cysts) accompanied by ovulatory dysfunction and excessive secretion of androgens [1]. Consensus on women health aspect of PCOS has suggested different criteria for diagnosis of PCOS in adolescents from those used for adults. According to its suggestions PCOS in adolescent should include all the three elements of Rotterdam criteria in which oligomenorrhea should be present after two years of menarche or primary amenorrhea at the age 16 years; polycystic ovaries on ultrasound along with ovarian size of more than 10 cm^3^ and hyperandrogenemia should be present [2]. The occurrence of polycystic ovarian syndrome has been associated with an increased risk for Type 2 diabetes, gestational diabetes, hypertension, and gynecological cancers [1, 3]. Studies have reported 10 times greater risk of developing Type 2 diabetes in women affected by PCOS [4]. The worldwide prevalence of polycystic ovarian syndrome ranges from 2.2 to 26% [4]. The rates of polycystic ovarian syndrome have been reportedly high among Indian women compared to their Caucasian counterparts [5], with an estimated prevalence of 9.13% in Indian adolescents [4, 6].

Clinical presentations of polycystic ovarian syndrome include abnormal facial and skin hair growth (hirsutism), acne, and irregular, or absence of, menstrual periods [7]. However acne is most common during adolescent phase of life and there is limited literature on adolescent androgenic alopecia [2]. Differential diagnosis of PCOS includes congenital adrenal hyperplasia (late onset) hyperthecosis, Cushing syndrome, hyperprolactinemia, hypothyroidism, and ovarian and adrenal androgen secreting tumors [2]. Different categories in the clinical presentations of PCOS have been distinguished according to the Rotterdam criteria [7]. They include (i) “classic PCOS” characterized by the presence or absence of ovarian cysts with excessive androgen secretion and irregular menstrual periods, (ii) “ovulatory PCOS” characterized by the presence of increased androgen secretion and multiple cysts, and (iii) “nonandrogenic PCOS” associated with irregular menstruation and multiple cysts [7].

The determinants of polycystic ovarian syndrome have been linked to both hereditary and environmental factors [1]. The attributed hereditary factors include early age of sexual maturation, premature fetal development, and family history of PCOS among first-degree relatives [1, 8]. Studies have reported an earlier age at diagnosis of PCOS (9–12 years) among adolescent females with earlier maturation of sexual characteristics compared to their later counterparts (13–18 years) [8]. This has been attributed to an increased androgen secretion associated with early onset of puberty [1]. It has been reported that premature fetal development leads to an earlier and more rapid onset of puberty with an increased risk of developing PCOS [9]. Clinical manifestations of associated symptoms such as hyperinsulinemia have also been observed in offspring of PCOS affected women long before the onset of puberty affirming the role of family history [9].

The associated environmental factors reported include physical inactivity, obesity, and its associated insulin resistance [7]. Insulin resistance which is of high prevalence in the Indian population [7] has been consistently reported as a strong determining factor for the occurrence of PCOS in Indian adults and adolescents [1]. While several studies have reported an association between excessive androgen secretion and the occurrence of insulin resistance in affected women, temporality has not been established [1]. There are marked variations in the prevalence of insulin resistance across different geographical regions of India and among urban and rural settings [10]. A higher prevalence of insulin resistance has been observed in urban Indian populations compared to their rural counterparts [10]. This is suggestive that a marked difference could exist in the prevalence of PCOS among different settings.

Since the clinical manifestations of PCOS have been consistently observed in early adolescence, the increased risk of developing Type 2 diabetes and its associated comorbidities during later years can be controlled by identifying high risk populations and implementing preventive measures. However, the nature of the environmental and lifestyle determinants of PCOS including physical activity and obesity is suggestive of the fact that variations could exist in the prevalence of PCOS in urban and rural settings due to dissimilar dietary practices and the level of physical activity. Although studies have reported the prevalence of PCOS in Indian adolescents, no studies have examined the differences in prevalence rates in urban and rural settings. We hypothesize that the burden of PCOS will be considerably lower among rural Indian adolescents compared to their urban counterparts. Such results could foster the implementation of lifestyle preventive measures for PCOS and its associated comorbidities in different settings at an earlier stage. The aim of this study was to determine urban rural differences in the burden of polycystic ovarian syndrome among Indian adolescent females aged 12 to 19 years.

## 2. Materials and Methods

A pilot cross-sectional study was conducted for a period of one month (August-September 2013) at Balaji Hospital, Vellore, Tamil Nadu, India. The study participants enrolled constituted patients visiting this hospital from Vellore district, an urban setting, and other surrounding villages of Puliyankannu, Vanapadi, Lalapet, Chettithangal, and Vepoor, which are rural settings. A convenient based sampling was employed and the sampling frame used was the Outpatient Department of Balaji Hospital. The study participants enrolled constituted patients visiting this hospital from Vellore city (an urban setting) and other surrounding villages of Puliyankannu, Vanapadi, Lalapet, Chettithangal, and Vepoor, which are rural settings. The inclusion criteria of the study comprised of adolescent girls of age between 12 and 19 years having complaints of menstrual disorders and living in urban and rural settings respectively for at least past one year. Further these adolescent girls must have had attained menarche at least 2 years before their existing complaint of menstrual disorder and were ready to provide informed consent. Individuals not fulfilling the inclusion criteria or participating in other clinical trials or had mental and physical challenges or were not willing to participate in the study were excluded from study. Equal numbers of urban (*n* = 63) and rural (*n* = 63) participants were enrolled with a total of 126 participants. All participants were enrolled after obtaining informed consent or assent to participate in the study. All of the study participants were interviewed in a separate place from the clinician's office for maintaining participant's confidentiality. The study protocol was approved by the IRB of the Foundation of Healthcare Technologies Society, New Delhi (IRB#FHTS/025/2013). For maintaining the anonymity each participant was assigned unique identification codes.

### 2.1. Data Collection Tools

Information about the variables was gathered by using set of semistructured questionnaires and anthropometric assessment was done by using measuring tape, weighing scale, and standard height rod. Noninvasive sonographic scanning was done to identify polycystic ovaries. Further biochemical examination was done for obtaining the values of prolactin, testosterone, T_3_, T_4_, and TSH (thyroid stimulating hormone). Each section of data collection tool is summarized below.

*(i) Sociodemographics*. Information gathered included age (years), educational status, family structure, occupation, marital status, parity, family income, and work schedule [11]. Data was also gathered on the family history of chronic diseases.

*(ii) Anthropometric Measures.* Physical assessments involved multiple measurements on several anthropometric variables including (a) height (meters), (b) weight (kg), (c) waist circumference (cm), and (d) hip circumference (cm). All four variables were measured without footwear and with participant wearing light clothing. Height was measured by using higher standard rod and weight was measured using a weighing scale. BMI of individual participant was calculated by dividing weight (Kg) by height in square meter. Waist and hip circumferences were measured by using an inch tape. For waist measurement superior most part of hip bone was palpated and then measurement tape was encircled around the stomach just above this point and umbilicus anteriorly. Participants were asked to stand in relaxing position during the measurements. Hip circumference was measured by encircling the inch tape in broadest part of hip of the participants. Two readings of height, weight, waist circumference, and hip circumference were measured and their mean was considered as final value. Information regarding weight gain in the past three months was self-reported.

*(iii) Clinical History.* The study participants' current history of having disease conditions including diabetes, hypertension, dyslipidemia, anorexia, and hypothyroidism was gathered. Complaints of leukocoria,* Acanthosis nigricans* (skin pigmentation), and presence of acne were also self-reported.

*(iv) Menstrual History*. Several parameters assessed included bleeding duration per cycle, intensity of flow, frequency of cycle, menstrual disorders, and average interval between two consecutive periods, recent weight gain, and monthly regularity of flow [12]. Menstrual flow history was assessed by obtaining self-reported frequency of bleeding duration per cycle. The daily intensity of menstrual flow was characterized on a 5-point scale ranging from slight to heavy. A history of common menstrual disorders including oligomenorrhea, amenorrhea, polymenorrhea, and dysmenorrhea was self-reported.

*(v) Hirsutism/Androgen Production*. Hirsutism and androgen production were assessed based on the Ferryman-Gallewey model in which study participants selected their degree of hair growth across nine key anatomical sites based on pictographic representations [13]. The various anatomical sites included the lips, chin, chest, upper abdomen, lower abdomen, arms, thighs, upper back, and lower back. The severity of the condition was assigned based on a 4-point scale and individual scores for each anatomic site were allocated. A maximum score of 32 points was possible. The level of androgen production was classified into three categories including normal, mild, and moderate to severe, based on total scores. Normal androgen production was defined by a total score less than 8 points, mild androgen production by a total score ranging from 9 to 15 points, and moderate to severe production by a total score greater than 15 points. Hyperandrogenism (excessive androgen production) was characterized by average scores exceeding 8 points across all anatomic sites [13].

*(vi) Physical Activity and Diet Assessment.* Physical activity was self-reported based on the type of activity (i.e., walking, running, and playing football), intensity levels (minutes/hours/days), and the length of time spent on each activity [14]. The study participants' nature of work was assessed by obtaining information regarding the type of work, number of hours spent per day, and level of vigorous activity performed. A 24-hour dietary recall was conducted to assess the nutritional status of the subject. Participants were required to report their food consumption at various meal periods of the previous day. The food intake of the study participants was classified into various nutrition types based on the WHO (2001) food scoring technique [15, 16].

*(vii) Polycystic Ovary Assessment.* The presence of polycystic ovaries was confirmed using noninvasive, transabdominal ultrasonographic scanning [17]. A 3.5 MHz convex probe was employed in this process. Diagnosis for polycystic ovaries was considered positive if 12 or more follicles measuring 2 and 9 mm were found with ovary size of more than 10 cm^3^ [2].

*(viii) Biochemical Assessment.* For biochemical assessment venous blood sample was taken by trained staff in morning between 8 am and 10 am in early phase of cycle. Serum testosterone levels were obtained using the ELISA method [18]. Prolactin, T_3_, T_4_, and TSH level were assessed to rule out hyperprolactinemia and hypothyroidism.

*(ix) Individuals with PCOS.* Participants were evaluated for PCOS on the basis of NIH Criteria, Rotterdam criteria, and guidelines of Rotterdam Consensus on women health aspect of PCOS. Final diagnosis of PCOS was made if all three elements of Rotterdam criteria were present which included presence of oligomenorrhea after two years of menarche or primary amenorrhea at the age of 16 years, and polycystic ovaries on ultrasound along with ovarian size of more than 10 cm^3^ and hyperandrogenemia should be present. Hirsutism score of more than 8 was considered positive for hyperandrogenemia.

### 2.2. Statistical Analysis

Descriptive statistics were computed for various continuous and categorical variables and were reported as means, standard deviations, and percentage distributions. *T* statistics and chi-square statistics were used to compare means and percentage distribution among continuous and categorical variables. Data were analyzed using IBM SPSS statistical software version 22.

## 3. Results

### 3.1. Study Population Characteristics

The average age of the adolescent girls was 16 years (SD = 2) that lived mostly in a nuclear family structure (75%, *n* = 95) and were unmarried (100%, *n* = 126) and 28% (*n* = 35) of them had highest education up to some college. Average household income of the participants was 135,103 INR (US$ 2252 approximately). The average BMI of the participants was 21 Kg/m^2^ with 11% (*n* = 13) being overweight to obese. Twenty-six percent (*n* = 33) of the study participants reported a recent weight gain in the past 3 months. No history of chronic diseases was reported among these study participants. However, family history of diabetes mellitus and hypertension was reported (9%, *n* = 12).

### 3.2. Physical Activity and Dietary Pattern

Twenty-four percent (*n* = 30) of the study participants were involved in vigorous physical activity with 82% (*n* = 103) of them spending less than 30 minutes every day on any kind of physical activity. Majority of the participants are involved in other physical activities that included walking (51%, *n* = 64) and cycling (8%, *n* = 10).

**Figure 1:**
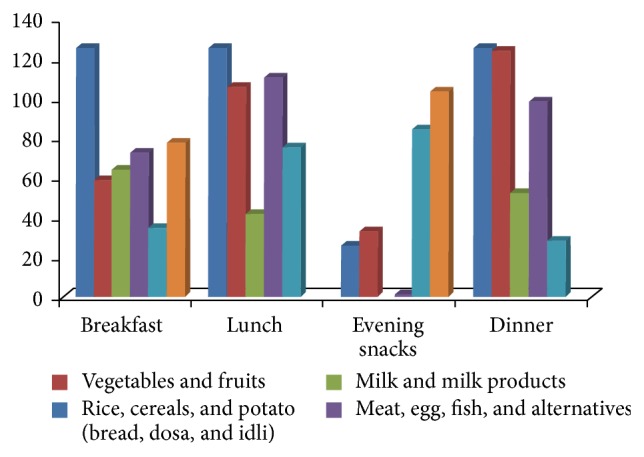
It depicts the frequency of daily reported food items consumed by the study participants.

Dietary intakes of cereals, fruits, vegetables, meat, egg, milk and its products, and fat and sweet food products were reported based on a 24-hour recall. Hundred percent of the participants reported consumption of cereals, potato, bread, and related food products during breakfast, lunch, and dinner. Consumption of vegetables and fruits was highest in dinner (99%; *n* = 125) followed by lunch (84%; *n* = 106). More than half of the participants reported consumption of milk or milk products in breakfast (51%; *n* = 64) followed by dinner (42%; *n* = 53). Overall 67% (*n* = 84) of the adolescents reported consuming fat and oil products in form of evening snacks along with tea (83%; *n* = 104). Meat, poultry, egg, and their alternatives were reported to be consumed highest during lunch time by 88% (*n* = 111) of the participants (Figure 1).

**Figure 2:**
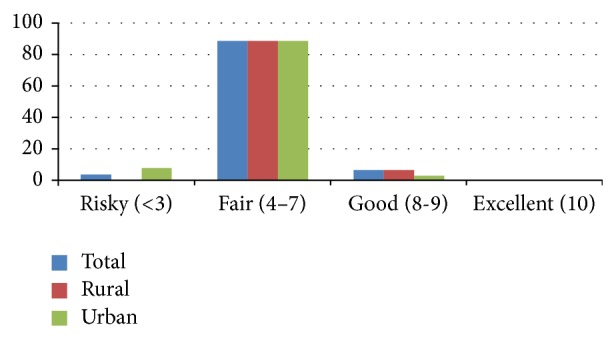
It depicts the food score categories among urban and rural participants.

The WHO food scoring technique was utilized in classifying the dietary intake of the various foods into four different groups including risky (<3), fair (4–7), good (8-9), and excellent (10) [15]. Eighty- nine percent (*n* = 112) of the study participants were classified as having a fair score. None of the participants received an excellent score (Figure 2).

### 3.3. Menstrual and Clinical History and Hirsutism

The average age at menarche was 12 (SD = 1) years. Oligomenorrhea (28%; *n* = 35) and irregular menses with weight gain (19%; *n* = 24) were the common menstrual disorders. The bleeding duration per cycle ranged from a minimum of 1-2 days to a maximum of 10–15 days. The presence of* Acanthosis nigricans* (skin pigmentation) was self-reported by 23% (*n* = 36) of the study participants and included sites such as thigh folds (13%, *n* = 16) and nape of the neck (9%, *n* = 12). The lips (92%; *n* = 116) and chin (68%, *n* = 86) were the common sites for hair growth with 48% (*n* = 60) having excess androgen production (hirsutism score of 9 and above).

### 3.4. Ultrasound and Blood Investigation

Sixty-three percent (*n* = 76) of the study participants had a positive radiology test reflecting presence of polycystic ovaries. Hundred percent of the participants have not sought any kind of treatment for polycystic ovaries. Average testosterone level of the participants was 2.47 pg/mL (SD = 1.6) ranging from .33 to 6.56 pg/mL. Thirty percent (*n* = 38) of the participants had above normal testosterone level. Participants average serum prolactin level was 14.7*µ*g/liter. Average values of T_3_, T_4_, and TSH were 152 ng/dL (SD = 23), 9.3*µ*g/dL SD = 5, and 2.8 *µ*U/mL, respectively (Table 1).

### 3.5. Characteristics of Study Participants in Urban and Rural Settings

A similar number of study participants were enrolled across urban (50%, *n* = 63) and rural (50%, *n* = 63) settings. There was minimal difference between the age of urban and rural participants. The proportion of participants living in nuclear family was higher among urban participants as compared to their rural counterparts (87% versus 63%; *P* = .002) and it was statistically significant. Family size, annual household income, and highest education level had not shown any statistically significant difference among the urban and rural participants. Thirty-three percent (*n* = 21) of participants in rural settings had some college education compared to their urban counterparts (22%, *n* = 14). Study participants in urban settings had a higher proportion of underweight adolescents (19%, *n* = 12), compared to those in rural settings (11%, *n* = 7). Average waist-hip ratio of rural participants (M = .85; SD = .03) was higher in comparison to urban participants (M = .84; SD = .05) and this difference was found to be statistically significant (*P* = .03). Nearly half (48%; *n* = 30) of the study participants in the urban settings reported a recent weight gain in the past three months, which was significantly higher compared to those in rural settings (5%, *n* = 3) (*P* < .0001). The average age at menarche for all the adolescent girls was similar for both urban and rural adolescents (mean = 12, SD = 1). Urban participants had a higher bleeding duration per cycle (M = 5 days; SD = 4) compared to their rural counterparts (M = 4 days; SD = 2) and this was statistically significant across both groups (*P* = 0.02). Frequency of less than or equal to 9 menses was higher among the urban participants in comparison to rural participants (67% versus 19%; *P* < .0001). Similarly urban participants (40%; *n* = 25) had higher proportion of individuals with oligomenorrhea in comparison with rural participants (16%; *n* = 10). Complaints of leucorrhea (24%, *n* = 15; *P* = .015) and weight gain with irregular menses (37%, *n* = 23; *P* < .0001) were also reported to be higher in frequency among urban participants. The urban study participants had a higher frequency of excess androgen activity (57%, *n* = 35; *P* = .001), which was evidenced by a higher occurrence of hair growth across several anatomical sites including the lips (99%, *n* = 62; *P* = .03), upper abdomen (72%, *n* = 45; *P* = .006), lower abdomen (60%, *n* = 38; *P* = .03), arms (75%, *n* = 47; *P* = .02), thighs (81%, *n* = 51; *P* = .001), and lower back (65%, *n* = 41; *P* = .01). Categorical distribution of hirsutism scores (*P* = .04) and positive ultrasonographic results for polycystic ovaries have shown statistically significant association with location of the participants. There was no statistically significant difference for serum testosterone level of the urban and rural participants. Engaging in vigorous work activity was significantly higher among rural study participants (38%, *n* = 24), compared to those in urban settings (10%, *n* = 6) (*P* < .0001). Frequency of good score on WHO food scoring technique was higher among rural participants (8%, *n* = 5) compared to the urban participants (3%, *n* = 2). The proportion of urban participants diagnosed with PCOS was higher than their rural counterparts (25% versus 11%; *P* = .03) (Table 1).

### 3.6. Association of Characteristics of Study Population with NIH Criteria and Rotterdam Criteria (Any of the Two Elements) and Consensus on Women Health Aspect of PCOS for Adolescents (Presence of All Three Elements) Suggestions

Analysis was performed to see any statistical significant association between the independent variables of the study participants with NIH criteria, Rotterdam criteria, and guidelines of Rotterdam Consensus for diagnosing PCOS. Twenty-five percent (*n* = 31) of the study participants were identified as having PCOS using the NIH criteria, compared to the 54% (*n* = 68) that were identified as having PCOS using the Rotterdam criteria (any of the two elements present). Eighteen percent of the participants were confirmed of having PCOS by recent guidelines of Rotterdam Consensus for adolescent diagnosis of PCOS (presence of all three elements). Majority of the individuals with PCOS had an average age of 16 (SD = 2) (*P* = .02) years with an average age of menarche 12 years (SD = 1). Results showed statistically significant association of Rotterdam Consensus (all three elements present) and weight gain in past three months (*P* = .006) and vigorous physical activity (*P* = .06) (Table 2).

## 4. Discussion

Adolescent phase of life brings multiple physiological, anatomical, and psychological changes in the life of girls. Due to familial, cultural, and societal restrictions most of the adolescent girls are not able to share and get right advice for menstrual related problems. PCOS is one of these conditions which is of serious concern. We have conducted this pilot study with the objective to determine the difference in burden of PCOS in urban and rural settings of India.

Results of the study have shown that 18% of participants were diagnosed with PCOS. Proportion of PCOS was higher in urban population in comparison to rural counterparts. Previous studies have reported a prevalence of PCOS ranging from 2.2 to 26% [19]. To the best of our knowledge, this is the second study which has been done to elucidate the proportion of polycystic ovarian syndrome among Indian adolescents. However, this study goes further by characterizing the distribution of the PCOS phenotypes and stratifying the proportion of participants based on urban and rural populations. The prior study reported a prevalence of 9.1% among 15–18-year-old adolescent females in Anantapur, Andhra Pradesh [4]. Average BMI of the study participants was similar to the prior study. However, our study had also reported the history of anorexia or bulimia (3%, *n* = 4) as well as a family history of diabetes mellitus (9%, *n* = 11), hypertension (9%, *n* = 12), and hypothyroidism (9%, *n* = 1). Previous studies in Italy, Iran, and Japan had shown 4.3%, 14.6%, and 26.2% prevalence of PCOS, respectively [19–21].

Involvement in vigorous work activity was significantly higher among rural participants compared to urban ones. Majority of the study participants 89% (*n* = 112) were classified as having a fair score (4–7) based on their dietary patterns and the distribution was similar among rural (89%, *n* = 56) and urban study participants (89%, 56). Having a fair score indicated that the participants needed some changes in their dietary pattern.

Despite the younger age of onset reported for polycystic ovarian syndrome, there is a scarcity of longitudinal studies examining the prevalence among adolescents. A study done in an urban Iranian population (*n* = 929) had a lower prevalence of excess androgen activity (22%, *n* = 205), compared to our population (48%, *n* = 60) [18]. Another prior study has shown 52.4% (*n* = 11) of the study participants having polycystic ovaries and 45.5% (*n* = 5) reported oligomenorrhea [21]. In our study the proportion of participants with polycystic ovaries and oligomenorrhea was 63% and 28%, respectively. None of the study participants was found positive for polycystic ovaries.

A major strength of this study is the wide range of adolescent population studied (12 to 19 years). However, the sample size (*n* = 126) utilized was considerably smaller compared to similar studies. Secondly it was a cross-sectional study so temporal association could not be determined. Further it was specialized hospital based study so its result cannot be generalized with the population. The increased risk of developing Type 2 diabetes as a result of polycystic ovarian syndrome is of immense public health concern especially in India, which has been tagged as the diabetic capital of the world [10]. The reported younger onset of this syndrome [8] and the prevalence of associated risk factors such as glucose intolerance [10] in the Indian population signify a need for intensified efforts in early detection. The increased proportion reported among urban adolescents compared to their rural counterparts is of great concern. Further studies should be conducted to elucidate the predominant risk factors in urban adolescents and the possible protective factors among rural adolescents in order to proffer preventive measures.

## Figures and Tables

**Table 1:** It shows sociodemographic, anthropometric, physical activity level, and menstrual problems of urban and rural participants.

Variables assessed	Rural % (*N*)	Urban % (*N*)	Total % (*N*)	*P* value
Age	Mean = 17; SD = 2	Mean = 16; SD = 2	Mean = 16; SD = 2	.50
Type of family				
Nuclear	63% (40)	87% (55)	75% (95)	.002
Joint	37% (23)	13% (8)	25% (31)	
Family size	Mean = 6; SD = 2	Mean = 5; SD = 1	Mean = 6; SD = 2	.13
Annual household income, INR	Mean = 135,682; SD = 28,725	Mean = 134,523; SD = 19,494	Mean = 135,103; SD = 24,456	.79
Highest education level of participant				
≤high school	19% (12)	27% (17)	23% (29)	.31
Intermediate (11th-12th grade)	48% (30)	51% (32)	49% (62)	
Some college	33% (21)	22% (14)	28% (35)	
Anthropometric characteristics				
BMI (kg/m^2^)	Mean = 21; SD = 3	Mean = 22; SD = 4	Mean = 21; SD = 3	.27
Underweight (<18.5)	11% (7)	19% (12)	14% (18)	.15
Normal (18.5–24.9)	83% (52)	67% (42)	71% (92)	
Overweight (25.0–29.9)	6% (4)	11% (7)	9% (11)	
Obese (30–34.9)		3% (2)	2% (2)	
Waist circumference (cm)	Mean = 76; SD = 9	Mean = 71; SD = 9	Mean = 73; SD = 9	.22
Hip circumference (cm)	Mean = 89; SD = 8	Mean = 84; SD = 8	Mean = 87; SD = 8	.20
Waist-hip ratio	Mean = .85; SD = .03	Mean = .84; SD = .05	Mean = .84; SD = .04	.03
Weight gain during past 3 months	5% (3)	48% (30)	26% (33)	<.0001
Physical activity, vigorous	38% (24)	10% (6)	24% (30)	<.0001
Menstrual history				
Age at menarche (years)	Mean = 12; SD = 1	Mean = 12; SD = 1	Mean = 12; SD = 1	.24
Bleeding duration per cycle (Days)	Mean = 4; SD = 2	Mean = 5; SD = 4	Mean = 5; SD = 3	.02
Frequency of cycle				
≤9 menses/year	19% (12)	67% (42)	43% (54)	<.0001
Menstrual disorders				
Oligomenorrhea	16% (10)	40% (25)	28% (35)	<.0001
Secondary amenorrhea	10% (6)	25% (16)	18% (22)	
Complaining of leucorrhoea	8% (5)	24% (15)	16% (20)	<.015
Irregular menses with weight gain	2% (1)	37% (23)	19% (24)	<.0001
Hirsutism (hair growth)				
Lips	86% (54)	98% (62)	92% (116)	.03
Chin	62% (39)	75% (47)	68% (86)	.24
Chest	51% (32)	60% (38)	56% (70)	.48
Upper abdomen	41% (26)	71% (45)	56% (71)	.006
Lower abdomen	43% (27)	60% (38)	52% (65)	.03
Arms	63% (40)	75% (47)	69% (87)	.02
Thighs	56% (35)	81% (51)	68% (86)	.001
Upper back	52% (33)	62% (39)	57% (72)	.32
Lower back	46% (29)	65% (41)	56% (70)	.01
Total hirsutism score criteria				
≤8 (normal)	60% (38)	45% (28)	52% (66)	.04
9–15 (mild)	35% (22)	38% (24)	37% (46)	
>15 (moderate to severe)	5% (3)	17% (11)	11% (14)	
Excess androgen activity	40% (25)	57% (35)	48% (60)	.001
Polycystic ovaries (ultrasound)	46% (29)	75% (47)	63% (76)	.05
Testosterone level (pg/mL)	M = 2.5; SD = 1.6	M = 2.4; SD = 1.5	M = 2.4; SD = 1.6	.77
Prolactin (*µ*g/L)	M = 15; SD = 3.3	M = 14.5; SD = 1.5	M = 14.7; SD = 3.2	.51
T_3_ (ng/dL)	M = 153; SD = 24	M = 150; SD = 22	M = 152; SD = 23	.43
T_4_ (*µ*g/dL)	M = 10; SD = 7	M = 8; SD = 3	M = 9.3; SD = 5	.08
TSH (*µ*U/mL)	M = 3; SD = 1.5	M = 2.5; SD = 1.5	M = 2.8; SD = 1.5	.09
NIH criteria				
Yes	14% (9)	35% (22)	25% (31)	.007
No	86% (54)	65% (41)	75% (95)	
Rotterdam criteria (any two elements of the total three)				
Yes	30% (19)	76% (48)	53% (67)	<.0001
No	70% (44)	24% (15)	47% (59)	
Rotterdam Consensus (all of the three elements)				
Yes	11% (7)	25% (16)	18% (23)	.03
No	89% (56)	75% (47)	82% (103)	

**Table 2:** It shows the demonstration of association between independent study variables with NIH and Rotterdam criteria (presence of any two or all three elements) of PCOS.

Variables assessed	NIH criteria % (*N*)		Rotterdam criteria (presence of any two of the three elements) % (*N*)		Rotterdam criteria (presence of all of the three elements) % (*N*)	
	Yes	No	Yes	No	Yes	No
Age (years)	M = 16 (SD = 2)	M = 17 (SD = 2)	M = 16 (SD = 2)	M = 17 (SD = 2)	M = 16 (SD = 2)	M = 17 (SD = 2)
	*P* = .016		*P* = .18		*P* = .020	
Type of family						
Nuclear	27% (26)	73% (69)	57% (54)	43% (41)	19% (18)	81% (77)
Joint	16% (5)	84% (26)	42% (13)	58% (18)	16% (5)	84% (26)
	*P* = .20		*P* = .12		*P* = .72	
Family Size	M = 5 (SD = 1)	M = 6 (SD = 2)	M = 5 (SD = 2)	M = 6 (SD = 2)	M = 5 (SD = 1)	M = 6 (SD = 2)
	*P* = .39		*P* = .60		*P* = .53	
Annual household income (INR)	M = 132903 (SD = 20403)	M = 135821 (SD = 25698)	M = 134794 (SD = 22518)	M = 135465 (SD = 26746)	M = 134000 (SD = 22401)	M = 135349 (SD = 24988)
	*P* = .56		*P* = .87		*P* = .87	
Highest education level of participant						
≤High school	41% (12)	59% (17)	59% (17)	41% (12)	31% (9)	69% (20)
Intermediate (11th-12th grade)	21% (13)	79% (49)	56% (35)	44% (27)	16% (10)	84% (52)
Some college	17% (6)	83% (29)	43% (15)	57% (20)	11% (4)	89% (31)
	*P* = .053		*P* = .34		*P* = .10	
Anthropometric characteristics						
BMI (kg/m^2^)	M = 22 (SD = 3)	M = 21 (SD = 3)	M = 22 (SD = 3)	M = 21 (SD = 2)	M = 22 (SD = 2)	M = 21 (SD = 3)
	*P* = .035		*P* = .51		*P* = .25	
Underweight (< 18.5)	10% (2)	90% (17)	5% (1)	95% (18)	1 (5%)	18 (95%)
Normal (18.5–24.9)	27% (25)	73% (69)	22% (21)	78% (73)	21 (22%)	78% (73)
Overweight (25.0–29.9)	27% (3)	73% (8)	9% (1)	91% (10)	1 (9%)	91% (10)
Obese (30–34.9)	50% (1)	50% (1)		100% (2)		2 (100%)
	*P* = .29		*P* = .63		*P* = .25	
Waist-hip ratio	M = .84 (SD = .04)	M = .84 (SD = .03)	M = .84 (SD = .04)	M = .85 (SD = .03)	M = .85 (SD = .03)	M = .84 (SD = .04)
	*P* = .87		*P* = .17		*P* = .17	
Weight gain during past 3 months						
Yes	42% (14)	58% (19)	88% (29)	12% (4)	36% (12)	64% (21)
No	18% (17)	82% (76)	41% (38)	59% (55)	12% (11)	88% (82)
	*P* = .006		*P* < .0001		*P* = .006	
Physical activity, vigorous						
Yes	7% (2)	93% (28)	16% (5)	84% (25)	7% (2)	93% (28)
No	30% (29)	70% (67)	65% (62)	35% (34)	22% (21)	78% (75)
	*P* = .009		*P* < .0001		*P* = .06	
Menstrual history						
Oligomenorrhea or amenorrhea	54% (31)	46% (26)	96% (55)	4% (2)	40% (23)	60% (34)
	*P* < .0001		*P* < .0001		*P* < .0001	
Age at menarche (years)	M = 12 (SD = 1)	M = 12 (SD = 1)	M = 12 (SD = 1)	M = 12 (SD = 1)	M = 12 (SD = 1)	M = 12 (SD = 1)
	*P* = .23		*P* = .49		*P* = .23	
Complaining of leucorrhoea						
Yes	25% (5)	75% (15)	65% (13)	35% (7)	15% (3)	85% (17)
No	24% (26)	76% (80)	51% (54)	49% (52)	19% (20)	81% (106)
	*P* = .96		*P* = .24		*P* = .76	
Prolactin (*µ*g/L)	M = 14.6 (SD = 3.3)	M = 14.8 (SD = 3.2)	M = 14.4 (SD = 3.1)	M = 15.1 (SD = 3.4)	M = 14.8 (SD = 3.3)	M = 14.7 (SD = 3.2)
	*P* = .72		*P* = .27		*P* = .84	
T_3_ (ng/dL)	M = 148 (SD = 22)	M = 153 (SD = 23)	M = 151 (SD = 23)	M = 152 (SD = 23)	M = 149 (SD = 21)	M = 152 (SD = 23)
	*P* = .32		*P* = .79		*P* = .51	
T_4_ (*µ*g/dL)	M = 10 (SD = 3)	M = 9 (SD = 6)	M = 9 (SD = 7)	M = 9 (SD = 2)	M = 10 (SD = 2)	M = 9 (SD = 6)
	*P* = .64		*P* = .89		*P* = .61	
TSH (*µ*U/mL)	M = 2.6 (SD = 1.6)	M = 2.8 (SD = 1.5)	M = 2.6 (SD = 1.5)	M = 3 (SD = 1.5)	M = 2.6 (SD = 1.6)	M = 2.8 (SD = 1.5)
	*P* = .53		*P* = .21		*P* = .44	
Testosterone (pg/mL)	M = 3.9 (SD = 1.5)	M = 1.9 (SD = 1.2)	M = 2.9 (SD = 1.6)	M = 1.8 (SD = 1.2)	M = 3.9 (SD = 1.5)	M = 2.2 (SD = 1.4)
	*P* < .0001		*P* < .0001		*P* < .0001	
Hirsutism score	M = 14 (SD = 3)	M = 6 (SD = 5)	M = 10 (SD = 5.2)	M = 6 (SD = 5.3)	M = 13 (SD = 3)	M = 7 (SD = 5.5)
	*P* < .0001		*P* < .0001		*P* < .0001	
≤8		100% (66)	25	41		66
>8	52% (31)	48% (29)	70% (42)	30% (18)	23% (38)	77% (37)
	*P* < .0001		*P* < .0001		*P* < .0001	
Polycystic ovaries (ultrasound)						
Positive	30% (23)	70% (53)	79% (60)	21% (16)	30% (23)	70% (53)
Negative	16% (8)	84% (42)	14% (7)	86% (43)		100% (50)
	*P* = .06		*P* < .0001		*P* < .0001	
